# Estrous cycle influences the expression of neuronal nitric oxide synthase in the hypothalamus and limbic system of female mice

**DOI:** 10.1186/1471-2202-10-78

**Published:** 2009-07-15

**Authors:** Monica Sica, Mariangela Martini, Carla Viglietti-Panzica, GianCarlo Panzica

**Affiliations:** 1 University of Torino, Department of Anatomy, Pharmacology and Forensic Medicine, Neuroscience Institute of Turin (NIT), Laboratory of Neuroendocrinology, , C.so M. D'Azeglio 52, 10126 Torino, Italy; 2 National Institute of Neuroscience-Italy (INN), Torino, Italy

## Abstract

**Background:**

Nitric oxide plays an important role in the regulation of male and female sexual behavior in rodents, and the expression of the nitric oxide synthase (NOS) is influenced by testosterone in the male rat, and by estrogens in the female. We have here quantitatively investigated the distribution of nNOS immunoreactive (ir) neurons in the limbic hypothalamic region of intact female mice sacrificed during different phases of estrous cycle.

**Results:**

Changes were observed in the medial preoptic area (MPA) (significantly higher number in estrus) and in the arcuate nucleus (Arc) (significantly higher number in proestrus). In the ventrolateral part of the ventromedial nucleus (VMHvl) and in the bed nucleus of the stria terminalis (BST) no significant changes have been observed. In addition, by comparing males and females, we observed a stable sex dimorphism (males have a higher number of nNOS-ir cells in comparison to almost all the different phases of the estrous cycle) in the VMHvl and in the BST (when considering only the less intensely stained elements). In the MPA and in the Arc sex differences were detected only comparing some phases of the cycle.

**Conclusion:**

These data demonstrate that, in mice, the expression of nNOS in some hypothalamic regions involved in the control of reproduction and characterized by a large number of estrogen receptors is under the control of gonadal hormones and may vary according to the rapid variations of hormonal levels that take place during the estrous cycle.

## Background

Nitric oxide (NO) is a messenger molecule, synthesized from arginine by a family of three distinct nitric oxide synthase (NOS) isoforms: (a) neuronal NOS (nNOS or NOS type I); (b) endothelial NOS (eNOS or NOS type III) that are Ca2^+ ^dependent; (c) macrophagical NOS (mNOS or NOS type II) that is Ca2^+ ^independent [1,2]. The neuronal isoform of this enzyme (nNOS), is regulated by several cofactors including calmodulin, and reduced nicotinamide adenine dinucleotide phosphate (NADPH) [3].

The NO system is implicated in the control of many behaviors [4]. In particular, several studies suggest that NO might facilitate the expression of rodents' sexual behavior in both sexes, at peripheral and central levels, probably through its action on specific neurotransmitter system (for a review see [5]). Peripherally, NO produced by eNOS and nNOS is involved in several tasks as the regulation of penile erection [6], ovulation [7,8] or transfer of oocytes from the ovaries to the oviducts [9]. Centrally, NO has an important role in controlling male and female sexual behavior [10-13]. This has been confirmed by the use of different lines of knockout mice. In particular the disruption of the exon 2 (resulting in residual nNOS activity due to the expression of alternatively spliced RNA forms) induces an improper sexual behavior [14], whereas the disruption of exon 6 (resulting in the total loss of nNOS activity) induces the disappearance of male sexual behavior and female ovulation [15]. Finally, NO promotes the release of gonadotropin releasing factor (GnRH) [16-18].

The control of reproductive behavior by NO is mediated at central level, by interactions with other neurotransmitter systems (for reviews see [19-21]). For instance, testosterone (T), in male rats, increases NO levels, through its action on nNOS. In the medial preoptic area (MPA), NO stimulates the release of dopamine (DA) that, in turn, promotes copulation [22]. The action on nNOS is mediated by the aromatization of T into 17β-estradiol (E_2_) and not by the reduction to dihydrotestosterone (DHT) [23]. Moreover, the NO-cGMP system is part of an intracellular signaling pathway to regulate the facilitatory effect of α_1_-adrenoreceptors on lordosis behavior in female rats (for a review see [24]).

A correlation between serotonin and NO is demonstrated by the partial alteration of the serotonin metabolism in mice lacking nNOS gene [25] and by the action of NO as a mediator of the stimulatory effects of serotonin in the MPA on luteinizing hormone secretion [26].

The presence of NO-producing neurons has been described in detail by means of histochemistry for NADPH-diaphorase, nNOS immunohistochemistry, and *in situ *hybridization, in numerous areas of the vertebrate central nervous system [27-34]. In the rat, the distribution of nNOS positive cells has been detailed in particular in hypothalamic and limbic nuclei involved in the control of the reproductive behavior: e.g., MPA, paraventricular (PVN), supraoptic (SON), arcuate (Arc) and ventromedial nucleus (VMH), bed nucleus of the stria terminalis (BST) [35-37], medial amygdala (MeA) and bed nucleus of the accessory olfactory tract (BAOT) [38,39].

Several studies in rodents show that, in these regions, nNOS expression is regulated by gonadal hormones. Castration decreases and treatment with T or its metabolite E_2 _increases the number of nNOS- or NADPH-diaphorase-positive cells, as well as the expression of mRNA for nNOS in MPA, PVN and ventrolateral part of the ventromedial nucleus (VMHvl) ([23,40-44]; for a complete bibliographic review see [5]).

The fundamental role of E_2 _and of estrogen receptors (ERs) in regulating/differentiating the nNOS system is also confirmed by our preliminary results obtained in mutant mice that either lacked the ERα receptor or were not exposed to E_2 _(aromatase knockout, ArKO) male mice. In both cases we detected a significant decrease of nNOS immunoreactivity in the MPA, PVN and Arc [45]. Moreover it has been shown that ERα and AR interact to regulate nNOS in male and female brain in a site-specific manner [46]. NADPH-diaphorase activity enhances during the estrus phase in the BAOT [39] and MeA [38] of female rat.

To further clarify the interrelationships between NO and gonadal hormones, we investigated here the effects of the estrous cycle on the expression of nNOS immunoreactivity in the hypothalamic and limbic system of female mice, with a focus on nuclei involved in the control of reproduction. In addition we have compared the distribution of nNOS immunoreactivity between male and female mice, in order to verify the presence of sexual dimorphism.

## Results

### Qualitative analysis

**Figure 1:**
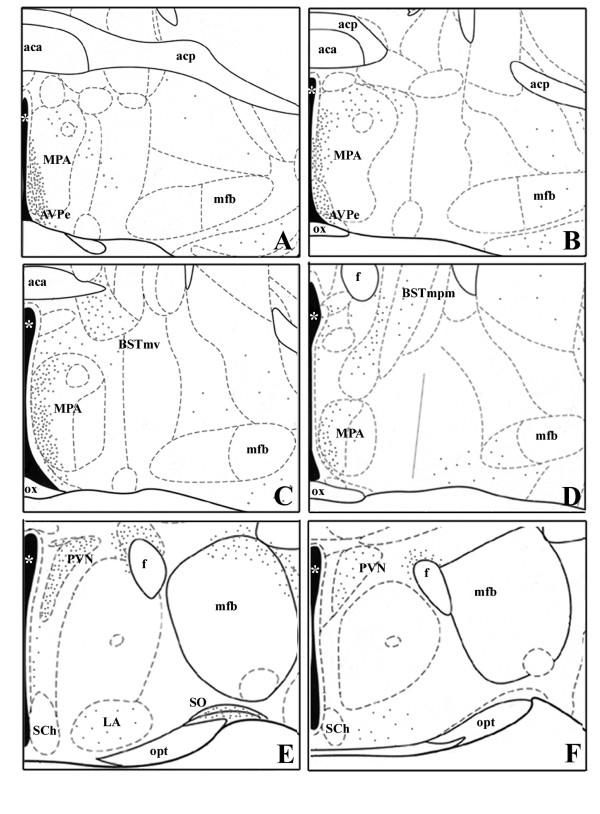
**A-F. Six drawings corresponding to coronal sections through a representative male mouse hypothalamus and limbic system arranged from the most rostral (A – preoptic area) to the most caudal (F – paraventricular region)**. Grey dots illustrate the distribution of nNOS-ir cell bodies. Nuclei are delineated according to the mouse brain atlas [69]. aca = anterior commissure, anterior; acp = anterior commissure, posterior; Arc = arcuate nucleus; AVPe = anteroventral periventricular nucleus; BSTmpm = bed nucleus of the stria terminalis, posteromedial subdivision; BSTmv = bed nucleus of the stria terminalis, ventromedial subdivision; DM = dorsomedial hypothalamic nucleus; f = fornix; LA = lateroanterior hypothalamic nucleus; mfb = medial forebrain bundle; ME = median eminence; MPA = medial preoptic area; opt = optic tract; ox = optic chiasm; PaAP = anterior parvicellular part of PVN; PaLM = lateral magnocellular part of PVN; PaV = ventral part of PVN; PVN = paraventricular nucleus; SCh = suprachiasmatic nucleus; VMH = ventromedial nucleus; VMHdm = ventromedial nucleus, dorsomedial part; VMHc = ventromedial nucleus, central part; VMHvl = ventromedial nucleus, ventrolateral part; * = third ventricle.

**Figure 2:**
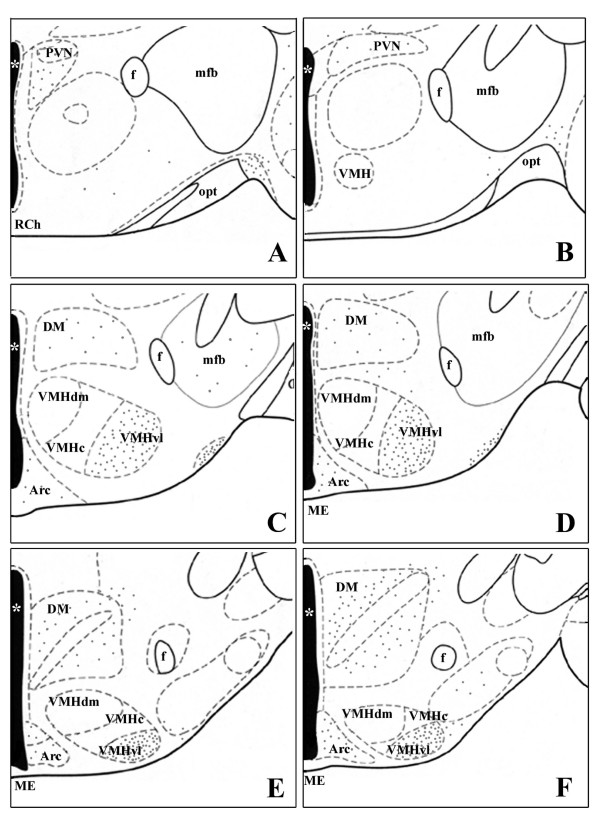
**A-F. Six drawings corresponding to coronal sections through a representative male mouse hypothalamus and limbic system arranged from the most rostral (A – paraventricular region) to the most caudal (F – posterior region)**. Grey dots illustrate the distribution of nNOS-ir cell bodies. Nuclei are delineated according to the mouse brain atlas [69]. aca = anterior commissure, anterior; acp = anterior commissure, posterior; Arc = arcuate nucleus; AVPe = anteroventral periventricular nucleus; BSTmpm = bed nucleus of the stria terminalis, posteromedial subdivision; BSTmv = bed nucleus of the stria terminalis, ventromedial subdivision; DM = dorsomedial hypothalamic nucleus; f = fornix; LA = lateroanterior hypothalamic nucleus; mfb = medial forebrain bundle; ME = median eminence; MPA = medial preoptic area; opt = optic tract; ox = optic chiasm; PaAP = anterior parvicellular part of PVN; PaLM = lateral magnocellular part of PVN; PaV = ventral part of PVN; PVN = paraventricular nucleus; SCh = suprachiasmatic nucleus; VMH = ventromedial nucleus; VMHdm = ventromedial nucleus, dorsomedial part; VMHc = ventromedial nucleus, central part; VMHvl = ventromedial nucleus, ventrolateral part; * = third ventricle.

The pattern of distribution of nNOS immunoreactive cells within the limbic-hypothalamic region of male mice was according to previous observations in rodents [30,32] and it is summarized in Figs 1 and 2.

**Figure 3:**
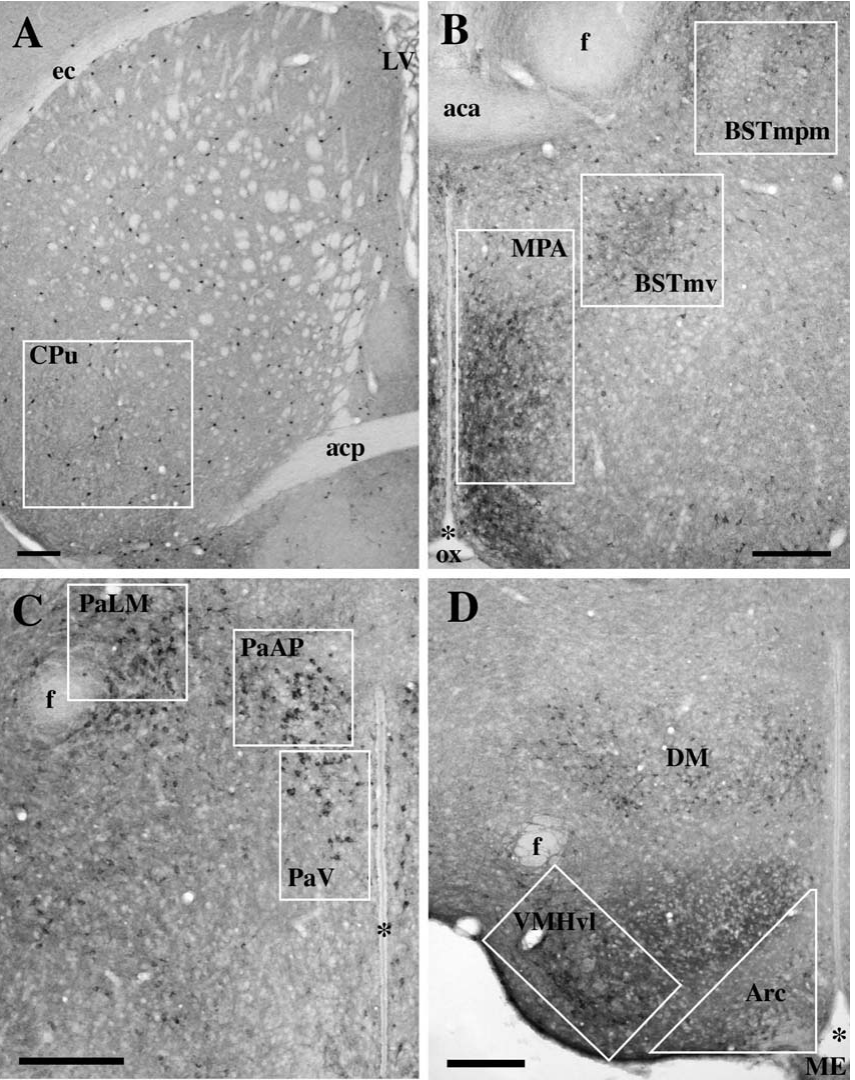
**Micrographs illustrating the nuclei considered in the experiment**. Boxed areas represent the regions in which nNOS-ir cells were quantified. Scale bar: 200 μm.

At the level of the anterior commissure (CA) a large number of positive neurons are found in the periventricular (AvPe) region and in the MPA ventral portion, close to the third ventricle. In the dorsal portion of MPA, only scattered, intensely stained neurons are present (Fig. 1A). They have a clear nucleus and an extended dendritic arborization. More caudally within the MPA, the nNOS-positive neurons are denser in the medial region than in the lateral, where, on the contrary, several positive fibers are present (Figs 1C, 3B).

A relevant group of nNOS-ir cells was observed in the BST, mainly clustered within its ventromedial subdivision (BSTmv); these cells were small, round, intensely or weakly stained with a relatively large nucleus (Figs 1C, 3B, Fig. 5).

A large number of intensely stained neurons are present in the PVN: these cells are mainly located in the dorsal part of the nucleus (Figs 1E, F, 2A, 3C), a large number of positive cells is located laterally to the PVN around the fornix. A cluster of intensely stained neurons is present also at the level of the supraoptic nucleus (Fig. 1E).

In the posterior hypothalamus, nNOS-ir elements are present in VMH and Arc nuclei. Within the VMH, intensely stained neurons are only present in the ventrolateral part of the nucleus (VMHvl, Figs. 2C, D, E, F). The other sub-regions of the nucleus are intensely stained due to the presence of a high number of positive fibers. In the Arc, round and weakly stained cell bodies are present in the whole extension of the nucleus (Figs. 2C, D, E, F, 3D). Scattered intensely positive cells are also present in the dorsal hypothalamic nucleus.

**Figure 4:**
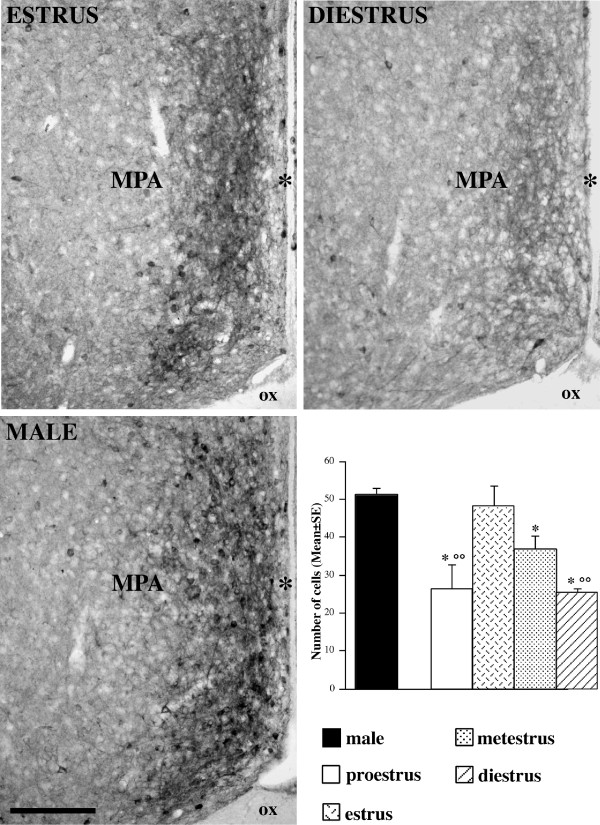
**Distribution of nNOS-ir neurons within the mouse medial preoptic area (MPA)**. Comparison among females in different stages of the cycle (estrus and diestrus) and males. Scale bar: 150 μm. The histogram reports the quantitative differences in the number of nNOS-ir cells (mean ± standard error). * = p < 0.05 in comparison to males; °° = p < 0.01 in comparison to estrus female.

**Figure 5:**
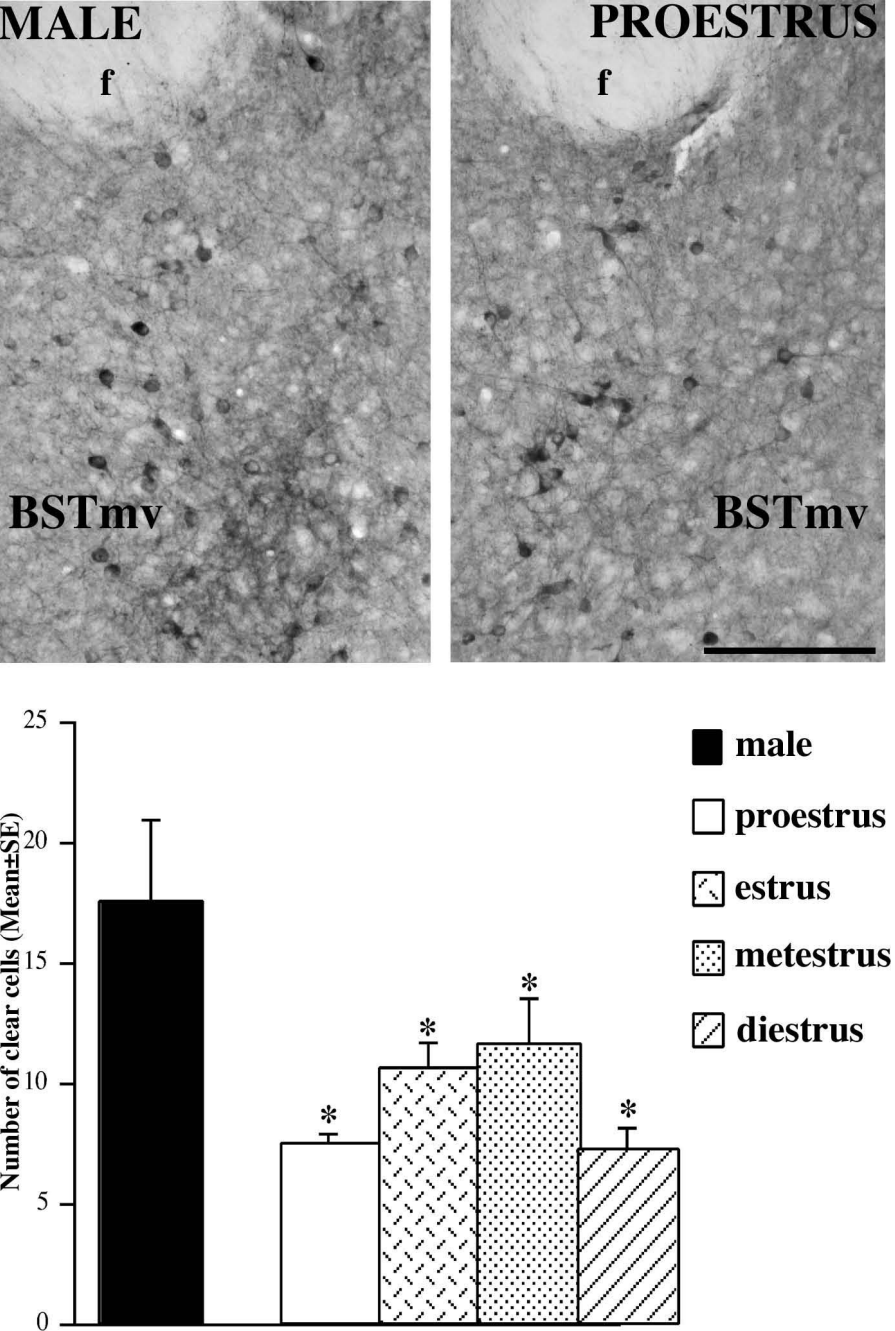
**Distribution of weakly stained (clear) nNOS-ir neurons within the mouse bed nucleus of the stria terminalis, ventromedial subdivision (BSTmv)**. Comparison among females and males. Scale bar: 150 μm. The histogram reports the quantitative differences in the number of nNOS-ir cells (mean ± standard error). * = p < 0.05 in comparison to males.

**Figure 6:**
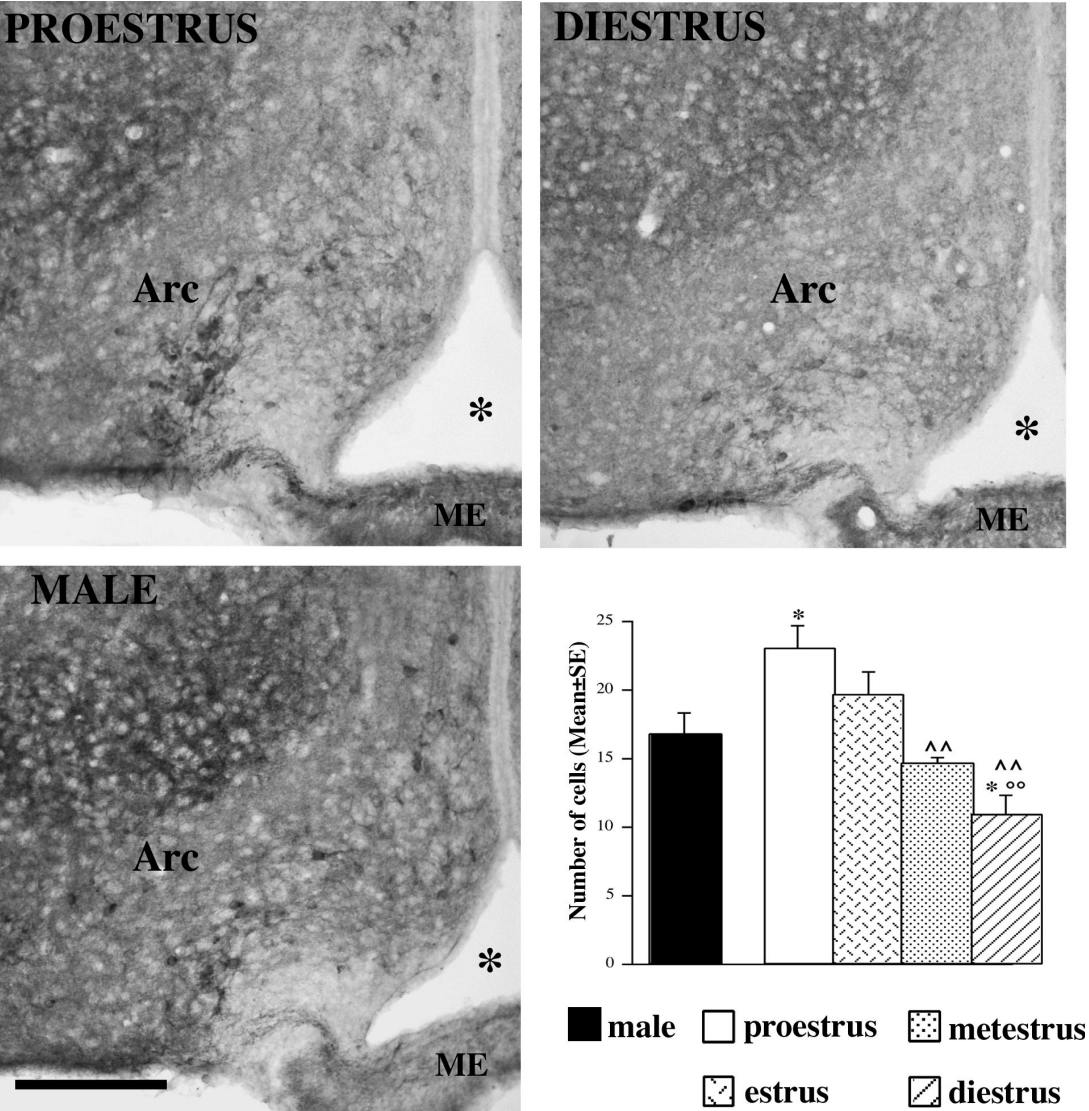
**Distribution of nNOS-ir neurons within the mouse arcuate nucleus (Arc)**. Comparison among females in different stages of the cycle (proestrus and diestrus) and males. Scale bar: 150 μm. The histogram reports the quantitative differences in the number of nNOS-ir cells (mean ± standard error). * = p < 0.05 in comparison to males; °° = p < 0.01 in comparison to estrus female; ^^ = p < 0.01 in comparison to proestrus females.

The observation of female mice taken at different stages of the estrous cycle evidenced a similar pattern of distribution, but at the same time suggested the existence of pronounced variations in cell number. Also the comparison with males evidenced some difference (Figs. 4, 5, and 6). To confirm these observations, we performed a quantitative analysis of the nNOS-ir elements.

### Quantitative analysis

The results of the quantitative analysis are summarized in Figs. 4, 5, and 6 [see also Additional File 1].

### Medial preoptic area (MPA)

In the MPA, the preliminary 2-way mixed ANOVA revealed not significant effect of the interaction among the cycle phases and the levels, therefore anterior-posterior levels were collapsed and the mean values of nNOS-ir cell number were analyzed by a one-way ANOVA. In this case, we found a statistically significant effect of the phase on the nNOS positive cell number (F_[4,20] _= 6.52; p = 0.002). The post-hoc Fisher PLSD test showed a significant decrease of positive cell number in proestrus (p = 0.004) and diestrus (p = 0.003) in comparison to estrus. Moreover, males had a higher number of positive cells than females in all the estrous phases with the exception of estrus (p = 0.001 for comparison with proestrus, p < 0.001 for comparison with diestrus, p = 0.04 for comparison with metestrus) (Fig. 4).

### Bed nucleus of stria terminalis, ventromedial and posteromedial subdivisions (BSTmv and BSTmpm)

In the posteromedial subdivision of the BST (BSTmpm), as well as in the ventromedial subdivision of the BST (BSTmv), the preliminary 2-way mixed ANOVA revealed not significant effect of the interaction among the cycle phases and the levels, and the following one-way ANOVA on the mean values of nNOS-ir cell number was also not significant.

However, we observed in both BSTmpm and BSTmv the presence of positive cells with a different intensity of immunostaining, that we have therefore classified as clear or dark positive elements. The one-way ANOVA for both clear and dark cells within the BSTmpm reported not significant differences for the cycle phase. A similar result was obtained with the one-way ANOVA for dark cells in the BSTmv. On the contrary, the one-way ANOVA for the number of clear nNOS-positive cells within the BSTmv reported a significant effect of cycle phase (F_[4,20] _= 4.03, p = 0.015). The Fisher PLSD test revealed that this effect is due to a higher number of positive clear cells in males in comparison to females in proestrus (p = 0.002), estrus (p = 0.027), and diestrus (p = 0.002) (Fig. 5).

### Arcuate nucleus (Arc)

In the Arc the preliminary 2-way mixed ANOVA revealed not significant effect of the interaction among the cycle phases and the levels, therefore anterior-posterior levels were collapsed and the mean values of nNOS-ir cell number were analyzed by a one-way ANOVA. In this case, we found a statistically significant effect of the cycle on the nNOS positive cells number (F_[4,20] _= 7.04, p = 0.001). The post-hoc Fisher PLSD test showed that proestrus and estrus have significantly higher numbers of positive cells than diestrus and metestrus (p = 0.0032 and p = 0.0001 for the comparison of proestrus versus metestrus and diestrus respectively; p = 0.0022 for the comparison of estrus versus diestrus). In addition, males showed significantly fewer positive cells than proestrous females (p = 0.021), and a significantly higher number of positive cells than diestrous females (p = 0.027) (Fig. 6).

### Ventromedial nucleus, ventrolateral part (VMHvl)

In the VMHvl the 2-way mixed ANOVA showed a significant effect of the interaction among the cycle phases and the levels (F_[1,4] _= 3.65, p = 0.022). Therefore, we performed the one way-ANOVA for each level. No effect of the phase was observed for the more rostral level (F_[4,20] _= 0.77, p = 0.55). On the contrary the one-way ANOVA for the more caudal level reported a value of p close to significance (F_[4,20] _= 2.55, p = 0.07). We have therefore applied the Fisher PLSD test, that reported significant differences in the number of positive cells among males and proestrous (p = 0.035), metestrous (p = 0.025) and diestrous females (p = 0.008), due to a higher number of positive cells in males in comparison to females.

### Paraventricular nucleus (PVN) and Caudate-Putamen

No significant variations were detected in the PVN [7]or CPu during estrous cycle nor in the comparison male-female [see Additional File 1].

**Figure 7:**
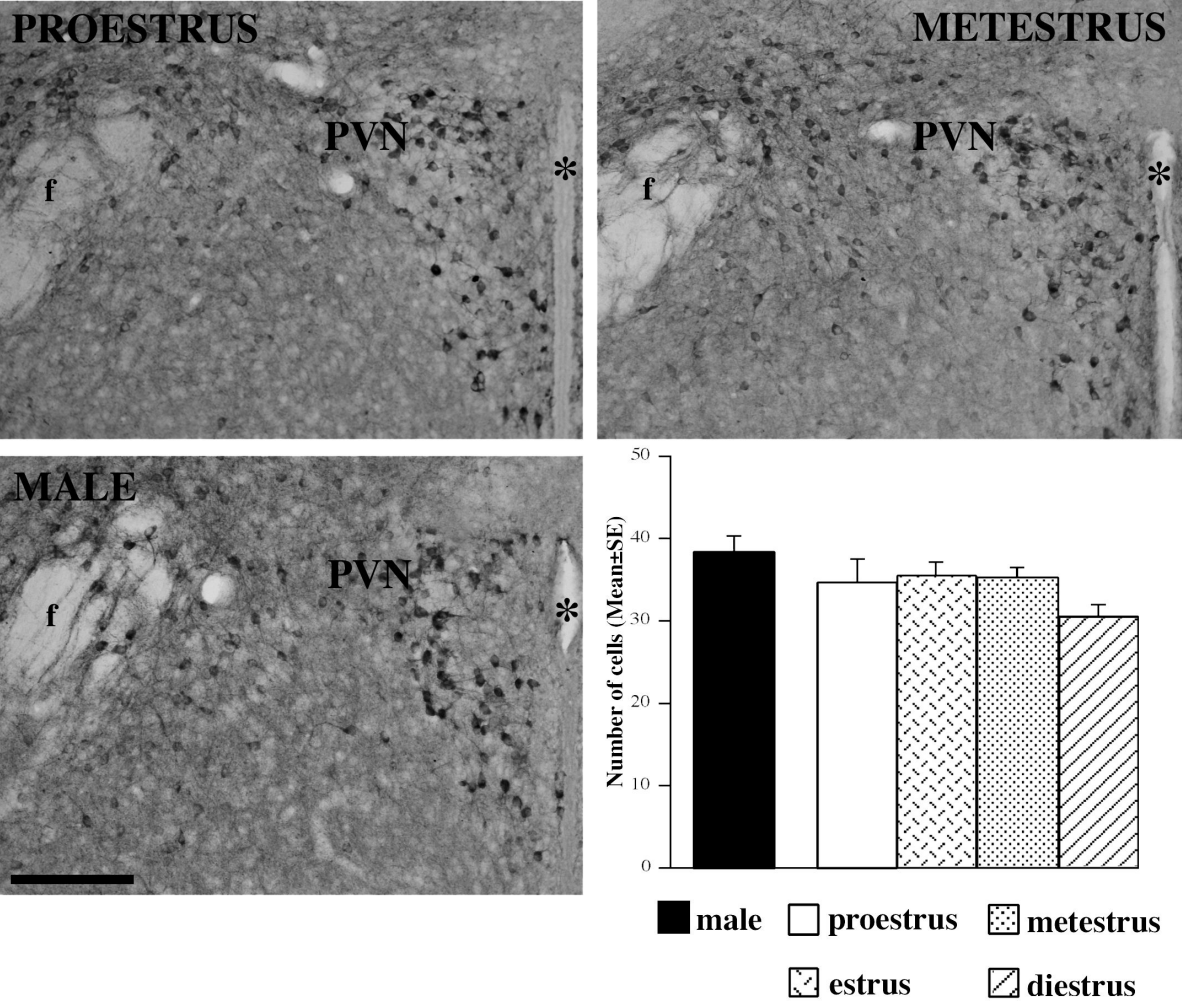
**Distribution of nNOS-ir neurons within the mouse paraventricular nucleus (PVN)**. Comparison among females in different stages of the cycle (proestrus and metestrus) and males. Scale bar: 150 μm. The histogram reports the mean number of nNOS-ir cells (mean ± standard error) per each examined stage.

## Discussion

Present data show that during the estrous cycle the expression of nNOS may significantly vary in some limbic-hypothalamic nuclei. Statistically significant changes in the nNOS-ir cells number were observed only in MPA and Arc, whereas in the other nuclei (e.g., PVN, BSTmpm, BSTmv and VMHvl) the NOS-ir cells number does not show significant changes during the different stages of the estrous cycle. Moreover, changes in the number of nNOS-ir cells in MPA and Arc do not follow the same pattern. In MPA, the highest number of positive neurons was detected during estrus, whereas in proestrus and diestrus we have the lower values. In Arc, the highest number of nNOS-ir cells was detected in proestrus, while metestrus and diestrus show the lower number of NOS-ir cells.

The comparison with males shows a stable sex dimorphism (males have a higher number of nNOS-ir cells in comparison to almost all the different phases of the estrous cycle) in the VMHvl and in the BSTmv (when considering only the less intensely stained elements, e.g. probably the part of NO-producing population that is less active). In MPA and Arc, sex differences were detected only comparing some phases of the cycle. In both nuclei, females in estrus have a number of positive cells that is not significantly different with that of males.

The distribution of nNOS immunoreactive elements within the limbic-hypothalamic region confirms previous reports in both mice [30,47] and rats [32]. Major clusters of NO-producing neurons are present within the preoptic area, the BST, the PVN and the VMH; scattered positive elements are present in the Arc nucleus. All these regions show, in mice, a large number of alpha and beta estrogen receptors (ER) [48,49], progesterone receptors (PR) [50], and androgen receptors (AR) [51], therefore confirming the idea that gonadal hormones may modulate the expression of nNOS [5].

### Effects of estrous cycle on the nNOS-ir distribution and male-female comparison

As reported in the introduction, the majority of previous experimental studies have been performed after long exposure to high levels of gonadal hormones (in particular testosterone or estradiol) [40,52] and only a few studies have addressed if and how nNOS expression is physiologically regulated. In particular, the nNOS system of some telencephalic nuclei of the accessory olfactory pathway (bed nucleus of the accessory olfactory tract and anteroventral subdivision of the medial amygdala) shows a significant increase in NADPH-diaphorase positive cell number in estrous females in comparison to diestrous ones [38,39]. Also the levels of cGMP (NO second messenger) are influenced by the estrous cycle: the release of cGMP in the MPA increases in the proestrus and diestrus afternoon in female rats [53].

In the present study, two hypothalamic nuclei related to the control of reproduction (MPA and Arc) show variations in the nitrinergic neuronal population during the estrous cycle. However, in the same phase of the cycle, the proestrus (when the estrogens reach their peak), we observed the highest level of positive elements in Arc and the lowest in MPA. The NO system of the MPA is target for the action of gonadal hormones also in males; in fact long-term castration induces a decrease, and treatment with testosterone induces an increase in the number of nNOS-ir cells in male rats [41] and male Syrian hamsters [42].

In the other investigated nuclei (i.e., PVN, VMHvl, BSTmpm, BSTmv), even though a large amount of estrogen receptors are expressed, we have not observed any significant change in the NOS-ir distribution during the female estrous cycle. The lack of effects in the PVN is particularly surprising. In fact, several studies in rodents have documented changes in the expression of nNOS or NADPH-diaphorase following changes in E_2 _availability. Administration of E_2 _increases the number of NADPH-diaphorase positive cells in male rats [43], whereas ERα KO or ArKO male mice showed a significant decrease in NOS-ir cell number in PVN [54,55]. One obvious remark is that the studies referred to were performed in males, while data in females that lack ER or aromatase (KO mice) or have constant exogenous E_2_, do not show significant hormonal regulation of NADPH-diaphorase [43]. In addition, the use of NAPDH-diaphorase histochemistry for the identification of nNOS system can introduce some discrepancies. In fact, both in the rat hypothalamus [56] and in the mouse basal forebrain [57] the number of nNOS-ir cells was higher than that of NADPH-diaphorase positive elements.

A second important remark is that, in the female, even the short-term treatment of ovariectomized individuals (an experimental situation that should emulate the estrus) results in an exposure to high levels of E_2 _for several days, while the natural conditions during the estrous cycle is that the peak of E_2 _is lasting only for a few hours, therefore the experimental simulation is probably overstimulating the system. In our opinion, this explains the lack of changes that we have observed at the level of VMH during the cycle, in contrast to the significant increase of nNOS mRNA in VMH [40,58] observed after short-term (2 days) or long-term (8-days) E_2_-treatment of ovariectomized female rats. We believe that this difference is the result of the very short term and transient changes happening during the estrous cycle compared to the effects of a longer exposure. In addition, in these previous experiments the effect of E_2 _only has been considered, whereas in natural conditions, all the gonadal hormones (including progesterone) are changing.

Obviously, when such significant variations take place, also the comparison with males may vary according to the stage of the cycle. Therefore, in MPA sexual differences are significant for proestrus, metestrus and diestrus (with the nNOS expressing cells more numerous in males than in females), suggesting the existence of activational effects, rather than a stable dimorphism of this system. In Arc proestrous and diestrous females have higher numbers of NOS-ir elements in comparison with male mice.

### Colocalization of NOS-ir elements with gonadal hormones' receptors

Estrogen, androgen and progesterone receptors co-localize with nNOS in rodent hypothalamus, however different studies evidenced different degrees of co-existence in rats and mice. In MPA 25–50% of nitrinergic elements express ERα in male rat [59,60], and 90% of them in male mouse [46]; whereas 50% of nNOS cells express AR in male rat [60] and only 20% in male mouse [46]. In anteroventral periventricular nucleus 60% of nNOS cells are co-localized with ERα, and 77% are co-localized with AR in male rat [60]. In VMH about 90% of nitrinergic elements co-localize with ERα in male rat [59]. ERα co-localize with 16% and AR with 6% of nNOS cells in BST, while 10% of nitrinergic neurons co-localize with ERα or AR in PVN of male mouse [46]. Furthermore, nitrinergic elements and PR show a low degree of co-localization in the MPA (6–16%) in comparison to VMH (55–57%) of the guinea pig [44]. In addition, in MPA fluctuations of ERα and PR expression accompany the hormonal events that occur during the rat estrous cycle [61]. Summarizing, these data suggest that ERα is an important factor to induce the expression of nNOS within the limbic and hypothalamic system, but, due to the differences in the co-localization, its importance may vary among the different nuclei of these regions. This is also confirmed by studies using mice lacking ERα, AR or both [46,54] that demonstrated that ERα may upregulate nNOS expression in a nucleus-specific way.

### Gonadal hormone-mediated control of nNOS expression

Present data and those reported in the literature clearly indicate a relationship among changes of estrogen levels and expression of nNOS, however, several problems are still unsolved. At first, the majority of data presented in the literature were taken in experiments involving one sex (generally the male), and in very different hormonal conditions (gonadectomy, short or long hormonal treatment, genetic manipulations). In addition, the situation in the rat could be strongly different from the mouse: about 50% of nNOS neurons in male rat MPA colocalize with ERα [60], whereas 90% of nitrinergic neurons co-localize in male mouse MPA [46], and corresponding data are not available for females of both species.

Examining our data, the most surprising feature is that the apparent patterns of estrous cycle influences are not the same across neuronal cell groups. We believe that a multifactorial hypothesis should be considered in order to explain this fact. At first, as summarized before, different nuclei have a different degree of co-localization among nNOS cells and gonadal hormones' receptors. These differences may determine the different degrees of stimulation or depression of the nNOS system in the examined nuclei.

Secondly, the expression of ERα across the estrous cycle varies independently in the different hypothalamic nuclei [62]. This has been observed in female rats, demonstrating that estrogen receptor mRNA levels vary during the estrous cycle, but the magnitude and direction of change observed during the cycle is region specific. This suggests that factors, other than endogenous estrogen levels, may differentially modulate ER mRNA expression in the hypothalamus. Among these factors, the selective estrogen receptor modulators (SERMs) may have a prominent role. For example, some nuclear receptor co-activators, as the Steroid Receptor Coactivator-1 (SRC-1), control ER and PR action in brain and affect distinct aspects of hormone dependent sexual behaviors [63]. Moreover, SRC-1 expression change in a region-specific manner in the rat brain during the estrous cycle [64]. This is also the case of the transcription factor AP-2 [65,66].

In addition to an indirect role on the expression of nNOS, through differentially regulating ER expression, SERMs may have a direct influence as well. In fact, the promoters of nNOS gene in mouse and in humans are regulated by some of these SERMs or transcription factors. Thus, in human nNOS gene the sequence inspection of 5'-flanking regions revealed the presence of AP-2 [67], whereas the steroidogenic factor 1 (SF-1), regulates nNOS gene expression in rat pituitary gonadotropes [68].

The functional implications of these changes in nNOS immunoreactivity are probably as complex as the putative mechanisms that are influencing them. In particular, several studies demonstrated that higher levels of nNOS imply an increased production of NO and the stimulation of its intracellular signaling pathway culminating in an increase of cGMP (for a review see [24]). Therefore, it is possible that fluctuations in the amount of nNOS within defined nuclei (MPA, ARC or, for a limited extent, BST) may relate to activation/inactivation of NO signaling pathway. Unfortunately, whereas a limited amount of information about changes in cGMP levels around the estrous cycle are available for female rat [53], similar data are not known at this time for female mouse and, due to the strong differences in co-localization of ERα and nNOS in rats and mice (see before), it seems not viable to speculate that cGMP changes will appear in the same direction also in female mouse. More experimental data are necessary to understand how the hormonally induced changes in nNOS may influence endocrine and/or behavioral parameters.

## Conclusion

In summary, the data collected in this study show and confirm the presence of nitrinergic neurons in several hypothalamic nuclei involved in the control of reproductive activity of male and female mice. In addition, we demonstrated that in female mice there are at least two populations of NO producing neurons, sensitive and insensitive to gonadal hormones fluctuations during the estrous cycle. The mechanisms of this differential, site-specific, sensitivity are not clear, but probably are based on the different pattern of distribution of AR, ER, and PR and SERMs within the nNOS system.

## Methods

### Animals

A total of 25 mice (5 males and 20 females) were employed in the study. We used a mouse strain, which was previously obtained in our laboratory by crossing two lines of mice: C57BL/6 and DBA2. Animals were housed, in monosexual groups of five, in cages with free access to food and water and maintained on a 12 h light 12 h dark cycle at a temperature of 23 ± 2°C.

The females were inspected for the stage of the estrous cycle (examination of a single vaginal smear) immediately before the sacrifice, and they were divided into 4 groups [proestrus (n = 5), estrus (n = 5), metestrus (n = 5), diestrus (n = 5)], depending on the day of the cycle.

Animal care was in accordance with the European Community Council Directive of November 24, 1986 (86/609/EEC), and the experimental protocol was approved by the Ethical Committee of the University of Torino and the Ministero dell'Università e della Ricerca Scientifica e Tecnologica.

### Fixation and tissue preparation

At the age of two months the mice were sacrificed using anesthesia overdose and perfusion. The animals were subjected to an anesthesia overdose (intraperitoneal injection of tri-bromo-ethanol, 250 mg/kg), followed by trans-cardiac perfusion of a saline solution (0.9%), until the return blood was clear, and then of 150 ml of fixative [4% paraformaldehyde in 0.1 M phosphate buffer (PB), pH 7.3–7.4].

Brains were dissected out of the skull, post-fixed for 2 h at 4°C in the same fixative, and rinsed in 0.01 M phosphate buffered saline (PBS). They were then placed overnight in a 30% sucrose solution in PBS, frozen in liquid isopentane at -35°C, and stored in a deep freezer at -80°C until sectioning.

Brains were serially cut in the coronal planes at 25 μm thickness with a cryostat. The plane of sectioning was oriented to match the drawings corresponding to the coronal sections of the mouse brain atlas [69]. Sections were collected in a cryoprotectant solution [70] at -20°C. Every fourth section (a section every 100 μm) one was processed for nNOS immunohistochemistry after 24 h washing in PBS. Adjacent sections were Nissl-stained with toluidine blue, or used for controls. Brain sections obtained from different experimental groups were stained simultaneously in order to reduce as much as possible between assays variances.

### nNOS immunohistochemistry (IHC)

The sections were stained for nNOS by the avidin-biotin method according to our standard procedure [30]. Briefly, to block endogenous peroxidase activity, sections, collected in multidish wells, were immersed in a solution of methanol/hydrogen peroxide for 20 minutes. After washing, they were incubated overnight at room temperature with an anti-nNOS rabbit antibody (DiaSorin, MN, USA, diluted 1:12.000 in PBS, pH 7.3–7.4, containing 0.2% Triton X-100). The antigen-antibody reaction was revealed by the biotin-avidin system (BAS, Vectastain Elite kit, Labtek). The peroxidase activity was visualized with a solution containing 0.15 mg/ml 3,3'-diamino-benzidine (Sigma, Milano, Italy) and 0.025% hydrogen peroxide in 0.05 M Tris-HCl buffer pH 7.6. Sections were collected on chromalum-coated slides, air-dried, washed in xylene and coverslipped with Entellan (Merck, Milano, Italy).

The commercial antibody against nNOS was generated in rabbit against a C-terminal synthetic peptide sequence (1419–1433) of human nNOS. The manufacturer (dr. Jeffrey Spangenberg, IncStar, Stillwater, MN) tested the specificity of the antibody by Western blot analysis and pre-adsorption with synthetic human nNOS (5 mg per ml of antibody at working dilution). No cross reactivity with other forms of NOS was reported [71,72]. The nNOS antiserum has been successfully used in human, rat, mouse, cat and monkey tissue. In particular, the specificity of this antibody for mouse central nervous tissue was tested in nNOS knockout mice where cerebellar and amygdala staining was totally abolished [73]. In our material, we have performed the following controls: a) the primary antibody was omitted or replaced with an equivalent concentration of normal serum (negative controls); b) the secondary antibody was omitted. In these conditions, cells and fibers were totally unstained.

### Quantitative analysis

Quantification procedure was performed according to our previously published method [74]. Briefly, in the present study we assessed seven different limbic-hypothalamic nuclei identified on the basis of the stereotaxic mouse brain atlas [69]. For each animal two standardized sections of comparable levels per nucleus were examined: medial preoptic area (MPA, corresponding to Bregma 0.02 mm and -0.10 mm), ventrolateral part of the ventromedial nucleus (VMHvl, corresponding to Bregma -1.46 mm and -1.58 mm), arcuate nucleus (Arc, corresponding to Bregma -1.46 mm and -1.58 mm), posteromedial (BSTmpm) and ventromedial (BSTmv) subdivision of the bed nucleus of the stria terminalis (corresponding to Bregma 0.02 mm and -0.10 mm), paraventricular nucleus (PVN, corresponding to Bregma -0.58 mm; -0.82 mm; -1.06 mm) and caudate-putamen nucleus (CPu, corresponding to Bregma 0.02 mm, only one section examined).

For the analysis of the PVN nNOS-ir elements distribution, we assessed 3 sections containing the nucleus. The positive elements were investigated within 3 main subdivisions: the ventral part (PaV); the lateral magnocellular part (PaLM) and the anterior parvicellular part (PaAP).

Positive neurons were identified for the presence of clearly labeled cell body and proximal processes. Manual cell counting was performed with a Leitz Laborlux microscope, equipped with a camera lucida, by using ×10 objective. Within the BST we observed the presence of positive cells with a different intensity of the cytoplasmatic immunostaining (the nucleus was always unstained). We have therefore subjectively classified as clear cells those exhibiting a cytoplasmic weak signal, whereas the other elements characterized by an intense cytoplasmic staining were classified as dark cells (Fig. 5). This subjective criterion was confirmed by density analysis performed on a few samples utilizing optical density measurement with Image J program on digitized images. This analysis demonstrated that neurons classified as dark cells show an intensity of the reaction at least double than those classified as weak cells.

For each level of the analyzed nuclei we selected a frame with a standardized area (500,000 μm^2 ^for MPA; 140,000 μm^2 ^for BSTmpm, BSTmv and VMHvl; 120,000 μm^2 ^for each subdivision of the PVN; 110,000 μm^2 ^for Arc; 540,000 μm^2 ^for CPu) and counted the immunoreactive cells within this frame (Fig. 3).

To detect changes in the nNOS-ir cell number during the female cycle and in comparison to males, we performed a two-way mixed ANOVA with cycle phase plus males as between subjects variable and anterior-posterior level as within subject (repeated) variable. When we detected the presence of a significant effect of the interaction phase-level we performed a separate one-way ANOVA for each level. When preliminary analyses revealed no significant effects of anterior-posterior levels, the level was collapsed and the average number (calculated using the average values from two sections) was analyzed with 1-way ANOVA. Differences were considered statistically significant for values of p < 0.05. These analyses were followed, when appropriate, by Fisher PLSD test, using the software Statview 5.0 (Abacus Concepts, Berkely, CA, USA).

Sections were photographed with a Zeiss Axioplan I microscope, equipped with a Nikon Coolpix 990 digital photocamera connected to an Apple G4 Macintosh. Digital images were processed using Adobe Photoshop 7.0 (Adobe Systems Incorporated, San Jose, CA, USA).

## Abbreviations

AVPe: periventricular region; AR: androgen receptor; Arc: arcuate nucleus; ArKO: aromatase knockout mice; BAOT: bed nucleus of the accessory olfactory tract; BST: bed nucleus of the stria terminalis; BSTmv: bed nucleus of the stria terminalis, ventromedial subdivision; BSTmpm: bed nucleus of the stria terminalis, posteromedial subdivision; CPu: caudate-putamen nucleus; cGMP: guanosine 3',5'-cyclic monophosphate; DA: dopamine; DHT: dehydrotestosterone; E_2_: 17 β-estradiol; ERα KO: estrogen receptor α knockout; ER: estrogen receptor; GnRH: gonadotropin releasing hormone; MeA: medial amygdala; MPA: medial preoptic area; NADPH: reduced nicotinamide adenine dinucleotide phosphate; nNOS: neuronal nitric oxide synthase; NO: nitric oxide; PaAP: anterior parvicellular part of PVN; PaLM: lateral magnocellular part of PVN; PaV: ventral part of PVN; PR: progesterone receptor; PVN: paraventricular nucleus; SO: supraoptic nucleus; T: testosterone; VMH: ventromedial nucleus; VMHvl: ventromedial nucleus, ventrolateral part.

## Authors' contributions

MS and MM designed and carried out the experiment, analyzed the data and wrote the paper. CVP and GCP coordinated the work, designed the experiment, analyzed the data and wrote the paper. All authors read and approved the final manuscript.

## Supplementary Material

Additional file 1 **Mean number of nNOS-ir cells (± standard error) in different nuclei, in different phases of the estrous cycle, and in males.**. Mean number of nNOS-ir cells (± standard error) in different nuclei, in different phases of the estrous cycle, and in males. In the right column F and p values of the one-way ANOVA. In bold significant (or close to significant) values. * = p < 0.05 in comparison to males. ° = p < 0.05 in comparison to estrus females. ^^ = p < 0.01 in comparison to proestrus females Click here for file
