# Supplementary material for: Estrous cycle influences the expression of neuronal nitric oxide synthase in the hypothalamus and limbic system of female mice
Source: BMC Neurosci. 2009 Jul 15;10:78. doi: 10.1186/1471-2202-10-78 (PMC2717099; doi:10.1186/1471-2202-10-78)
Supplement: Additional file 1 — Mean number of nNOS-ir cells (± standard error) in different nuclei, in different phases of the estrous cycle, and in males.. Mean number of nNOS-ir cells (± standard error) in different nuclei, in different phases of the estrous cycle, and in males. In the right column F and p values of the one-way ANOVA. In bold significant (or close to significant) values. * = p < 0.05 in comparison to males. ° = p < 0.05 in comparison to estrus females. ^^ = p < 0.01 in comparison to proestrus females [file 1471-2202-10-78-S1.doc]

|  |  |  |  |  |  | |  |
| --- | --- | --- | --- | --- | --- | --- | --- |
|  |  |  |  |  |  | |  |
| **Nucleus** | **Males** | **Proestrus** | **Estrus** | **Metaestrus** | **Diestrus** | | **ANOVA** |
|  |  |  |  |  |  | |  |
| **MPA** | 50.80+2.45 | 26.10+6.69* ° | 47.80+6.17 | 36.40+4.26* | 25.10+1.36* ° | | **p<0.001** |
| **BSTmv** | 33.00+3.32 | 27.50+1.44 | 35.10+3.17 | 32.80+4.91 | 24.80+2.65 | | n.s. |
| **BSTmpm** | 31.00+5.75 | 26.50+7.13 | 30.60+2.82 | 37.20+7.51 | 31.30+6.26 | | n.s. |
| **BSTmv-dark cells** | 15.60+1.22 | 20.20+1.40 | 24.70+2.81 | 21.40+3.50 | 17.70+1.59 | | n.s. |
| **BSTmv-clear cells** | 17.40+3.66 | 7.30+0.64* | 10.40+1.45* | 11.40+2.11* | 7.10+1.15* | | **p<0.05** |
| **ARC** | 16.60+1.89 | 22.80+1.99* | 19.50+1.95 | 14.40+0.83^^ | 10.70+1.80* ° ^^ | | **p<0.001** |
| **VMH-rostral** | 36.60+4.14 | 36.40+8.25 | 39.80+5.82 | 43.60+5.53 | 48.40+4.28 | | n.s. |
| **VMH-caudal** | 56.40+9.22 | 34.00+6.48* | 38.60+8.55 | 32.40+6.01* | 27.20+3.15* | | **p=0.071** |
| **PVN** | 45.90+4.11 | 47.10+4.39 | 45.70+3.37 | 51.30+3.34 | 43.20+2.78 | | n.s. |
| **PVN-PaV** | 13.60+2.23 | 10.20+2.70 | 9.60+1.43 | 11.20+1.77 | 14.00+1.76 | | n.s. |
| **PVN-PaAP** | 24.40+2.06 | 24.60+1.92 | 26.00+1.92 | 26.00+1.92 | 25.00+2.21 | | n.s. |
| **PVN-PaLM** | 9.20+2.51 | 8.20+0.80 | 8.80+3.23 | 13.20+2.92 | 8.00+1.89 | | n.s. |
| **CPu** | 21.60+1.66 | 19.00+0.55 | 21.40+36 | 17.00+1.14 | 21.20+1.66 | | n.s. |
|  |  |  |  |  |  | |  |
|  |  |  | |  | | |  |
| **TABLE 1** |  |  |  |  | |  |  |
|  |  |  |  |  | |  |  |
|  |  |  |  |  | |  |  |
|  |  |  |  |  | |  |  |
